# Editorial: Torquetenovirus: predictive biomarker or innocent bystander in pathogenesis

**DOI:** 10.3389/fmed.2023.1283454

**Published:** 2023-09-27

**Authors:** Tania Regina Tozetto-Mendoza, Paulo Henrique Braz-Silva, Simone Giannecchini, Steven S. Witkin

**Affiliations:** ^1^Laboratory of Virology, School of Medicine, Institute of Tropical Medicine of São Paulo, University of São Paulo, São Paulo, Brazil; ^2^Department of Stomatology, School of Dentistry, University of São Paulo, São Paulo, Brazil; ^3^Department of Experimental and Clinical Medicine, University of Florence, Florence, Italy; ^4^Department of Obstetrics and Gynecology, Weill Cornell Medicine, New York, NY, United States

**Keywords:** Torquetenovirus (TTV), TTV quantification, saliva, transplantation, sputum, pregnancy, amniotic fluid, aneurysm

Two decades ago, the existence of commensal viruses was revealed (1). It remains poorly understood how commensal viruses evade the host's innate and adaptive immune responses (2–5). Gene amplification and metagenomic analyses have highlighted Torquetenovirus (TTV) as a marker of immunocompetence and as a near universal component of the virome. This Editorial and the contributing articles provide unique data to motivate future investigations on TTV and its consequences for pathological conditions.

TTV is the most frequently identified member of the *Anelloviridae* family, a small, circular and single-strand DNA virus. The possible involvement of TTV in pathogenesis remains an unresolved issue. Does the presence or concentration of TTV initiate and/or potentiate a pathological change in susceptible populations? A second question of major clinical interest is whether the detection and/or measurement of the TTV titer in different biological fluids and tissues will be a clinically relevant biomarker of immune status and predictor of the presence or prognosis of specific disorders. This Research Topic presents new data relating to the clinical relevance of the TTV determination in diverse medical problems.

TTV quantification as a novel and sensitive method to assess immune status over long periods of time following organ transplantation is comprehensively assessed by Gore et al.. The current fragmentary state of knowledge of the immune response to TTV in healthy individuals is also reviewed. The advantages of TTV measurement for predicting short- and long-term outcome in organ transplant recipients are thoughtfully delineated.

Reactivation of a cytomegalovirus (CMV) infection is one of the major negative complications of solid organ transplantation. Prophylactic treatment of all transplant recipients with antiviral medication is not recommended due to adverse side effects. It would be beneficial to identify an assay that can select for treatment only those transplant patients who are at elevated risk for CMV infection. Mafi et al. present new data that determination of the TTV titer in the circulation at intervals up to one-year post-transplantation identifies individuals at highest risk for CMV viremia following kidney transplantation. The sensitivity of the TTV titer was comparable to commercial assays that measured the overall CD8+ lymphocyte cell-mediated immune response and the specific response to CMV antigens.

The regulation of pro-inflammatory immunity during gestation is essential for proper fetal development and carriage of the pregnancy to term. Immune system activation at term triggers term labor, while its activation at earlier stages of pregnancy is a major cause of preterm labor and delivery. Kyathanahalli et al. investigated plasma and saliva levels of TTV and the related virus, torquetenomidivirus (TTMV) in pregnant women in their second and third trimester in relation to pregnancy outcome, parity and race. There was no association between TTV titer in the second or third trimester and premature labor, but there was a significant association between the TTMV level in the second and third trimester and a lower gestational age at delivery and preterm birth. The TTV and TTMV titers were higher in women with a prior delivery than in those with a first pregnancy. Also, Black women had higher TTV and TTMV levels than did non-Hispanic white women. Although the value of TTV and TTMV analysis during gestation remains unresolved, their findings highlight that parity and race must be included as relevant variables in all analyses of TTV and TTMV levels, and this should not just be limited to pregnancy-related studies.

A second pregnancy-related study by Tozetto-Mendoza et al. measured TTV titers in amniotic fluid obtained from women undergoing *in utero* fetal surgery to correct a spinal defect in their fetus. While this surgery has better results than surgery performed after delivery, there is a higher rate of preterm birth. Of the 27 amniotic fluid samples available for analysis, three (11.1%) were positive for TTV. Unlike the great majority of TTV-related studies where TTV DNA is identified by polymerase chain reaction and not further characterized, in this study the TTV genomes were sequenced by metagenomic analysis to validate their identity and to determine their relationship to other TTV isolates. This study could serve as a blueprint for an expanded analysis of TTV that is detected in various biological samples. The presence of intraamniotic TTV was predictive of an earlier gestational age at delivery and also with the occurrence of respiratory distress in the newborns.

Three articles evaluated the value of TTV measurement at distinct sites linked to specific disorders. Xie et al. tested for TTV in sputum from adults suffering from chronic obstructive pulmonary disease (COPD). Almost every sample tested was positive for multiple TTV genotypes and the TTV titer correlated with COPD severity and decreased lung function. Falabello de Luca et al. quantitated TTV levels in plasma and saliva in patients with cirrhosis of the liver. They report that TTV was detected more than twice as often in saliva than in plasma, but there was no association between TTV and clinical parameters. Rabelo at al. evaluated the occurrence of TTV in the arterial wall of individuals with an intracranial aneurysm. Although no association was noted between aneurysm rupture and detection of TTV, the study validated that TTV can be detected in vascular tissue, possibly as a consequence of immune cell accumulation at an inflammatory site.

In conclusion, the seven studies reinforce the potential value of TTV measurement as a sensitive indicator of immune status in medically significant conditions. Whether TTV contributes to the observed immune system alterations and thus has a role in disease pathogenesis, or if its appearance at elevated levels is merely a secondary consequence of immune changes without added clinical significance remains undetermined.

## Author contributions

TT-M: Conceptualization, Data curation, Formal analysis, Supervision, Validation, Writing—original draft, Writing—review and editing. PB-S: Conceptualization, Formal analysis, Validation, Writing—review and editing. SG: Conceptualization, Formal analysis, Validation, Writing—review and editing. SW: Conceptualization, Data curation, Formal analysis, Supervision, Validation, Writing—original draft, Writing—review and editing.
